# Phosphate-solubilizing function of *Pediococcus pentosaceus* PSM16 and its underlying mechanism

**DOI:** 10.1128/spectrum.00491-25

**Published:** 2025-06-10

**Authors:** Hong Yaling, Chen Shasha, Li Mengyao, Li Wenhui, Gao Yan, Luo Mengjiao, Zhou Qian, Zhou Siyuan, Zhou Diao, Li Xinhui, Zhang Lei, Zhou Qiong, Yang Ziqiang, Xia Yandong, Zhang Gaihua, Jia Yin

**Affiliations:** 1 National and Local Joint Engineering Laboratory of Animal Peptide Drug Development, Hunan Provincial Key Laboratory of Animal Intestinal Function and Regulation, Hunan International Joint Laboratory of Animal Intestinal Ecology and Health, College of Life Sciences, Hunan Normal University 554899, Changsha, Hunan, China; 2 Ningxiang Fengyu Biotechnology Co.Ltd, Changsha, China; 3 Key Laboratory of National Forestry and Grassland Administration on Control of Artificial Forest Diseases and Pests in South China, Hunan Provincial Key Laboratory for Control of Forest Diseases and Pests, Key Laboratory for Non-Wood Forest Cultivation and Conservation of Ministry of Education, College of Life Science and Technology, Central South University of Forestry and Technology 659514 , Changsha, Hunan, China; Nanjing Agricultural University, Nanjing, China

**Keywords:** effective phosphorus, phosphatase, seed germination

## Abstract

Phosphorus is a crucial nutrient for plant growth, but only a limited quantity is typically accessible in the soil for plants to absorb directly. Phosphate-solubilizing bacteria (PSB) can convert inorganic phosphorus compounds into forms that are more readily usable for plant nutrition. Our previous research has verified the function of *Pediococcus pentosaceus* PSM16 in degrading phytic acid. On this basis, we further explored the phosphorus-solubilizing capacity of PSM16 and evaluated its potential for practical application in this study. The results indicated that PSM16 significantly enhanced phosphorus utilization, not only enriching the environment with bioavailable phosphorus but also lowering the environmental pH and conductivity. These changes are instrumental in enhancing soil fertility, providing favorable conditions for plant growth, and stimulating seed germination. Through whole-genome sequencing of PSM16, we have identified key genes associated with the production of acid phosphatase. Specifically, the genes of GM000834, GM000917, GM000925, and GM000974 are implicated in PSM16’s phosphorus solubilization function, likely through the production of phosphatase enzymes. Moreover, we have discovered that the phosphatases T.fus-QOS58989.1, A.cae-WP_156200763, M.the-SNW17984, N.gly-GGP12115, T.chr-SDQ48339.1, and T.chr-SDQ90039.1 are homologous to the aforementioned proteins and are present in compost, as confirmed by our informatics analysis. This presence in compost suggests their potential for real-world agricultural applications. This research presents promising candidate strains for the development of phosphorus-degrading bacterial agents, which could increase the efficiency of phosphorus fertilizers and contribute to sustainable agricultural practices. This strategy is not only effective but also environmentally benign and cost-effective, offering a valuable contribution to the field of agricultural biotechnology.

**IMPORTANCE:**

This study sheds light on the transformative power of the PSM16 strain, a paragon of phosphorus solubilization that adeptly converts inert phosphorus into a form that is readily absorbed by plants. In this way, it not only elevates the levels of available phosphorus in the environment but also enriches the soil fertility, supporting the healthy growth of plants. The strategic application of PSM16 in tandem with phosphorus fertilizers promises to enhance the utilization rates of these fertilizers, reinforcing sustainable agricultural initiatives and alleviating the environmental pressures caused by excessive application. In addition, the study has uncovered a trove of strains that hold promise for the development of safe dephosphorylating bacterial agents. These agents are poised to deliver an economical, efficient, and eco-friendly alternative, encapsulating a commitment to agricultural advancement that is both responsible and resourceful.

## INTRODUCTION

Phosphorus is one of the essential mineral elements for plant growth and development and plays a crucial role in the biosynthesis of various compounds such as phytoalexins, nucleic acids, and phospholipids (1). These include nucleic acid synthesis, membrane synthesis and stability, respiration, signaling pathways, enzymatic activities, and redox reactions (2). Phosphorus is the plant nutrient limiting factor that is second only to nitrogen in the extent of its impact on crop productivity and is one of the major factors limiting crop yields globally (3). The soil phosphorus reservoir encompasses both effective and ineffective forms, with the effective form being critical for plant nutrition, yet over 40% of global soils are low in this form (4).

In China, soils are short of soluble forms of phosphorus in most areas (5, 6), and reliance on non-renewable fertilizers is problematic (7). Upon application, much of soluble phosphorus becomes insoluble due to reactions with soil cations like Ca^2+^, Fe^3+^, Fe^2+^, and Al^3+^, making it inaccessible to plants. The seasonal efficiency of phosphorus fertilizers is also very low, typically between 5% and 25% (8–10). Additionally, excessive use of these fertilizers upsets the soil’s natural balance, causing acidification (11) and reduced fertility, which contributes to environmental pollution and contradicts sustainable farming practices.

Phosphorus-solubilizing bacteria (PSB) offer a solution by converting insoluble phosphorus into a soluble form for plants (12). This enhances soil health and plant growth, and when combined with chemical fertilizers, it results in sustainable improvement in soil fertility (13), showcasing their potential as a sustainable solution in agriculture. PSB can release organic acids (14) that chelate metal ions, reducing soil’s fixed phosphate and increasing its availability (15) or by liberating H^+^ to lower the pH value of the surrounding environment, and the fixed phosphate can then be converted into an accessible form for plants. In addition, PSB can secrete enzymes capable of catalyzing phosphatidylinositol mineralization. Phosphatase is one of the most commonly secreted enzymes by PSB (16), an enzyme that releases available phosphate from its substrate by dephosphorylation of phosphor anhydride or phosphodiester bonds in organic matter for dephosphorization.

Genomic analysis and metagenomic studies can expand our understanding of phosphate-solubilizing bacteria. A combination of metagenomic and amplicon sequencing methods can be used to investigate the microbial mechanisms of phosphorus cycling in soil, identifying the genes and proteins that play a role (17). Through bioinformatics analysis, we can start with identified phosphatase proteins and screen for homologous proteins that exist in actual production environment soils. Recently, some scholars have further developed a new single-cell technology that allows for the direct detection of the phosphate-solubilizing function of phosphorus-dissolving bacteria *in situ* in the soil (18), breaking through the limitations of genotypic research. Researchers can link the phosphate-solubilizing phenotype to specific bacterial groups and functional genes. This provides a powerful tool for exploring beneficial soil microorganisms and enhancing agricultural sustainability.

The study investigates the phosphate-solubilizing ability of PSM16 and its intrinsic mechanisms. The results showed that PSM16 is a highly efficient strain of phosphate-solubilizing bacteria, which can effectively increase the content of effective phosphorus in the environment, improve soil fertility, and promote plant emergence and growth. It can provide an effective economic means to improve the utilization rate of phosphorus fertilizer and the sustainable development of agriculture.

## RESULTS

### PSM16 can increase the amount of effective phosphorus in the environment

**Fig 1:**
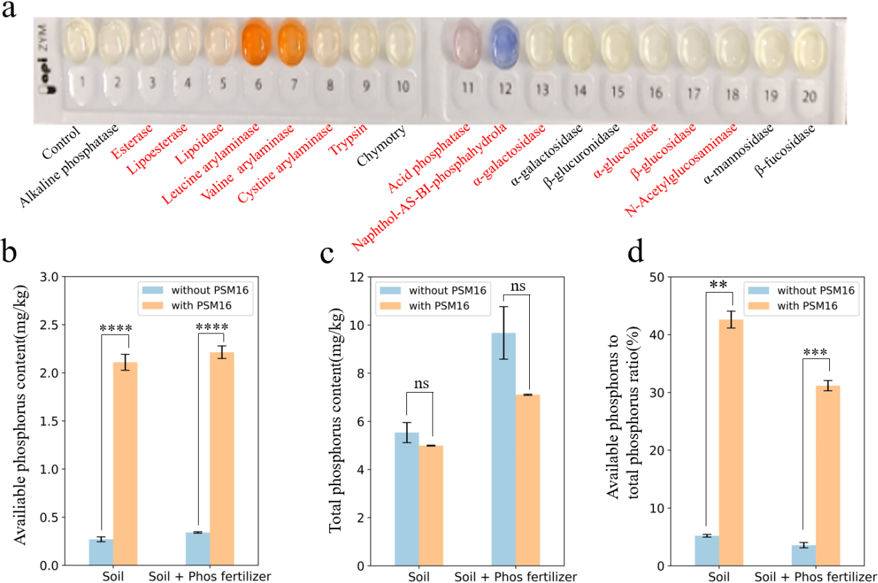
Effects of PSM16 on soil phosphorus levels (**a**) Acid phosphatase (S-ACP) activity of PSM16. (**b**) Total phosphorus levels in soil without and with PSM16. (**c**) Available phosphorus levels in soil without and with PSM16. (**d**) The Ratio of available to total phosphorus in soils with and without PSM16. Results are presented as mean values ± SD. *N* = 3. Statistical significance determined by *t*-test. *P < 0.05, **P < 0.01, ***P < 0.001, and ****P < 0.0001.

Our previous study screened *Pediococcus pentosaceus* PSM16 (19) from healthy sow milk, which can degrade phytic acid. This study found that PSM16 possessed acid phosphatase activity (Fig. 1a; Table S1). PSB can hydrolyze organic phosphorus in soil by secreting phosphatase and converting it into inorganic phosphate to achieve the purpose of phosphorus solubilization. To deeply investigate the specific effect of PSM16 on phosphorus in soil, we first cultivated PSM16 in soil. Following this, we measured effective and total phosphorus concentrations in the soil and assessed the utilization rate of phosphorus. The results showed that the content of effective phosphorus in the soil was significantly increased by adding PSM16 compared to the control (Fig. 1b), from 0.270 mg/kg to 2.109 mg/kg. A negligible change in total phosphorus (Fig. 1c), the percentage of effective phosphorus in total phosphorus was increased by nearly 40% (Fig. 1d). It is worth noting that the addition of phosphorus fertilizer to the soil without PSM16 did not increase the effective phosphorus significantly (Fig. 1b), with the content remaining at only 0.105 mg/kg. In contrast, adding PSM16 to the soil along with the phosphorus fertilizer led to a significant increase in effective phosphorus (Fig. 1b), while the total phosphorus showed a negligible change (Fig. 1c). The proportion of effective phosphorus to total phosphorus was also significantly higher (Fig. 1d).

In addition, we also investigated the effect of PSM16 on soil pH value and electrical conductivity, both of which are indicators of soil fertility. Different plants have different adaptations to soil pH values. The most suitable soil pH value for plant growth is usually between 5.2 and 6.8, and the soil conductivity that favors plant growth and development ranges from 350 to 1,000 μS/cm. We measured the pH value and conductivity of soil mixed with PSM16 bacterial solution, and the results showed that the pH value and the conductivity of the treated soil were within the range that is more suitable for plant growth, decreasing to 6.43 and 775.296 μS/cm, respectively. (Fig. S1).

The decrease in soil electrical conductivity is significantly correlated with increased acid phosphatase activity (20) and is also influenced by changes in soil properties like ion intensity and cation composition ratio (21), which can rapidly change with management and environmental conditions. Following the processing of the PSM16 bacterial suspension, we noticed a marked decrease in pH, which we hypothesize to be due to the secretion of organic acids. To validate this hypothesis, we analyzed the PSM16 fermentation broth to assess the production of organic acids by the PSM16 strain over its growth cycle. Our results revealed a positive correlation between cultivation time and the concentration of organic acids produced by PSM16 (Fig. S2). Notably, after 24 hours of cultivation, PSM16 was observed to produce significant quantities of acetic acid, propionic acid, valeric acid, isovaleric acid, and isobutyric acid (*P* < 0.05). By 48 hours, the strain significantly enhanced its production of butyric acid, acetic acid, propionic acid, valeric acid, isovaleric acid, and isobutyric acid, with butyric acid, acetic acid, and propionic acid being the most prevalent acids produced (Fig. S2). The production of these organic acids is likely due to the metabolic activity of PSM16, which utilizes carbohydrates and other carbon sources to produce these acids as byproducts of fermentation (14, 15). These organic acids not only lower the soil pH but also chelate metal ions, thereby increasing the availability of phosphorus for plant uptake (16).

Overall, PSM16 has a phosphorus-solubilizing function that increases the amount of effective phosphorus in the environment, which can lead to an increase in the bioavailability of phosphorus in the soil and can also affect soil fertility. This finding provides strong support for optimizing the use of fertilizers in agricultural practices to ensure that crops receive sufficient phosphorus for optimal growth while helping ameliorate the problem of overuse of phosphorus fertilizers and avoiding negative impacts on the environment.

### PSM16 promotes *Arabidopsis* germination

Given previous studies that have shown PSM16’s impact on the effective phosphorus content in soil, our hypothesis extends to its potential influence on seed germination and plant growth. To explore this, we conducted a focused investigation into the specific effects of PSM16 on the seed germination rate of the model plant *Arabidopsis thaliana*. We aim to achieve a more holistic understanding of its role in the growth and development of plants.

**Fig 2:**
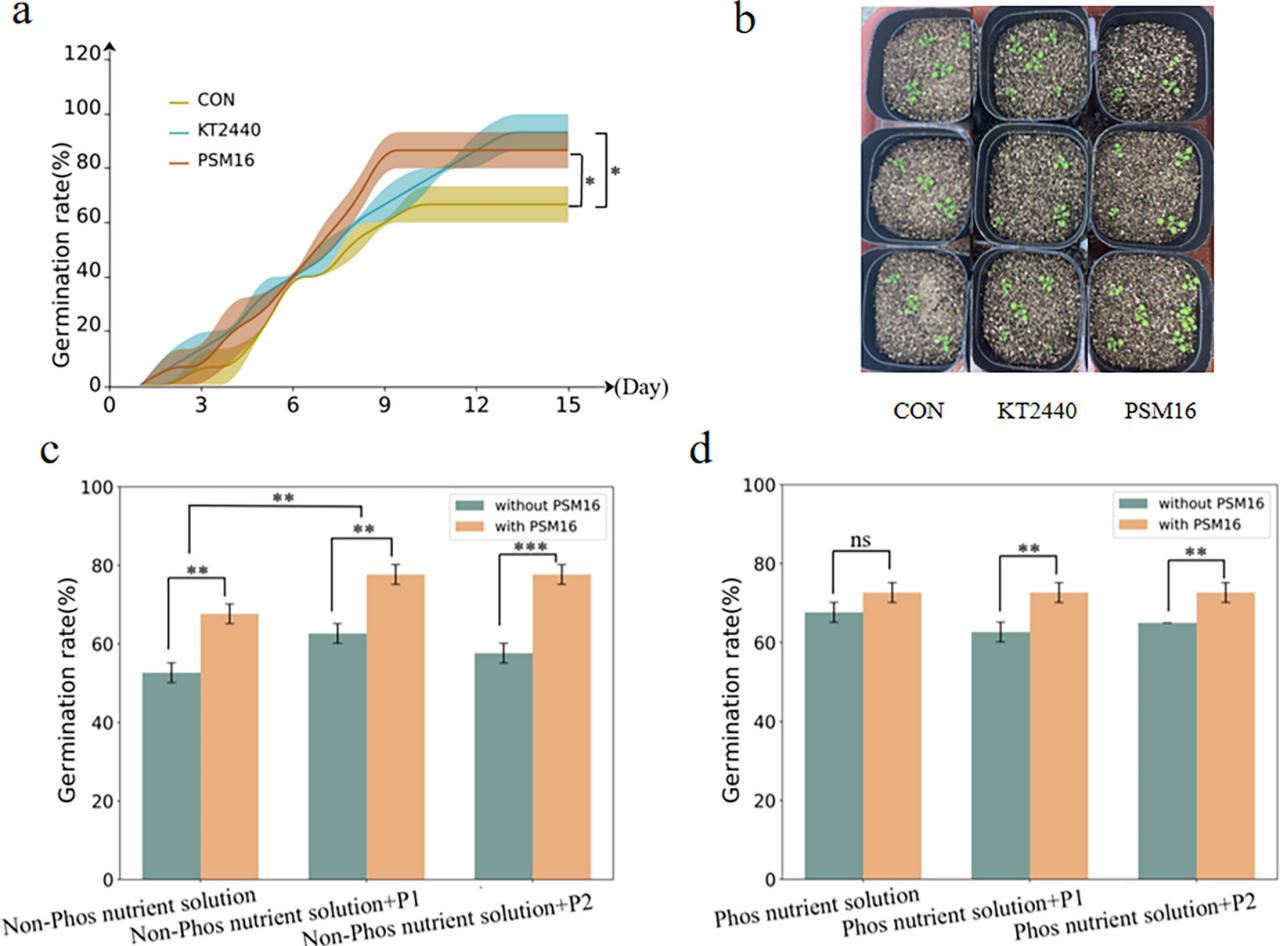
*Arabidopsis thaliana* germination rates in nutrient solutions with and without PSM16 and phosphorus. (**a**) The germination rate of *Arabidopsis thaliana* after the addition of KT2440 and PSM16. (**b**) *Arabidopsis thaliana* seedlings on day 15 post-addition of KT2440 and PSM16, showcasing their growth progression. The control image in this figure is the same as the control image in Fig. 4e as they are derived from the same experimental batch. This repetition reflects the consistency of the experimental conditions and is not an error. (**c**) The germination rate of *Arabidopsis thaliana* in a phosphorus-free nutrient solution with PSM16 was added on day 15. (**d**) The germination rate of *Arabidopsis thaliana* in a phosphorus-enriched nutrient solution with PSM16 added on day 15. Results are presented as mean values ± SD. *N* = 3. Statistical significance determined by *t*-test. *P < 0.05, **P < 0.01, and ***P < 0.001.

We selected *Pseudomonas putida* KT2440 as a positive control for plant growth promotion, given its well-documented role in enhancing seed germination through phosphate solubilization and rhizosphere interactions (22, 23). We conducted an experiment where *Arabidopsis thaliana* was cultivated in soil treated with either the PSM16 bacterial solution or the KT2440 solution. The control group has not been treated with the bacterial suspension. The results indicated that the PSM16 treatment yielded a germination rate of 86.67%, which was just 6.66% less than the 93.33% germination rate achieved with the KT2440 treatment. Both of these rates were markedly superior to that of the control group, which recorded a germination rate of 66.67% (Fig. 2a). Figure 2b further illustrates the robust growth of *Arabidopsis thaliana* post-germination, providing a visual representation of the treatments’ positive influence on seed germination and subsequent growth (Fig. 2b).

We further revealed that the addition of a certain amount of phosphorus fertilizer on top of the addition of a phosphorus-free nutrient solution could significantly enhance the seed germination rate. On this basis, comparing with the group without PSM16, we can find that the addition of PSM16 can significantly further improve the seed germination rate (Fig. 2c), which may be due to the addition of phosphorus fertilizer, which increases the total phosphorus content in the soil, and a larger portion of the ineffective phosphorus is converted into effective phosphorus by PSM16. This suggests that the addition of PSM16 can improve the utilization of phosphorus fertilizer, thus further improving seed germination.

When a phosphorus-free nutrient solution is added, the inclusion of PSM16 significantly enhances the germination rate of seeds (Fig. 2c). This further suggests that PSM16 can transform ineffective phosphorus into a form that seeds can absorb and utilize, thereby increasing the utilization rate of phosphorus in the soil and promoting seed germination.

The supplementation of PSM16 did not yield a significant improvement in seed germination when only a phosphorus-containing nutrient solution was utilized, likely due to the direct uptake of phosphorus by the plants from the solution itself. However, when varying amounts of phosphorus fertilizer were superimposed upon the phosphorus-enriched nutrient solution, the introduction of PSM16 led to a significant enhancement in the seed germination rate (Fig. 2d). This suggests that the additional phosphorus fertilizer contributed to an increase in the total phosphorus content of the environment, and PSM16 played a crucial role in converting the inert phosphorus within this total content into a form that is bioavailable to the plants, thereby promoting their growth.

In conclusion, the addition of PSM16 can improve the utilization of phosphorus in the soil and convert ineffective phosphorus into effective phosphorus, thus promoting seed germination.

### Mining of dephosphorylation genes

**Fig 3:**
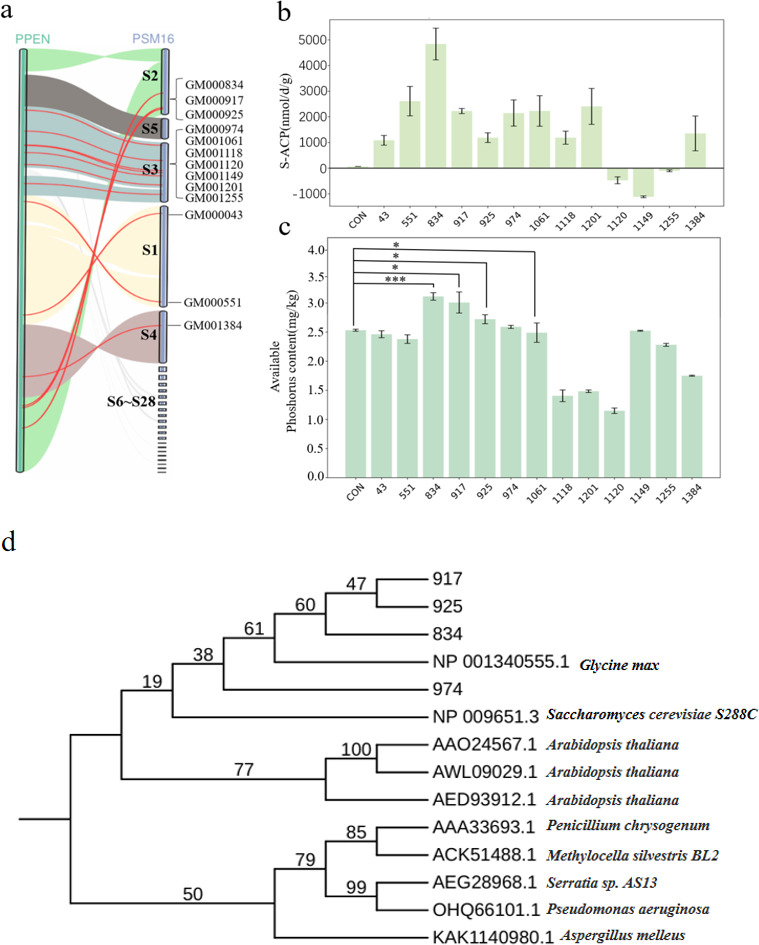
Mining of functional genes for dephosphorylation. (**a**) Identification of phosphatase-related genes within the PSM16 genome. (**b**) S-ACP activities of different *Bacillus subtilis* transformed with phosphatase genes. (**c**) Effect of different phosphatases on the content of soil effective phosphorus content. (**d**) A phylogenetic tree constructed with ten acid phosphatase proteins that have been previously reported and acid phosphatases 834, 917, 925, and 974. The accession numbers and the sources are shown next to the branches. Results are presented as mean values ± SD. *N* = 3. Statistical significance determined by *t*-test. *P < 0.05, **P < 0.01, and ***P < 0.001.

To delve into the molecular underpinnings of PSM16’s role in phosphatase conversion, we conducted a comprehensive genome-wide analysis to identify genes associated with phosphatase activity (Fig. 3a; Table S2). Subsequently, we individually transformed *E. coli* with expression plasmids harboring these genes. The experimental results showed that, compared to the control (CON), the strains transformed with the gene of GM000834, gene of GM000917, gene of GM000925, and gene of GM000974 genes not only exhibited a significant increase in acid phosphatase (S-ACP) activity (Fig. S3a), but also led to a marked increase in available phosphorus in the soil treated with these engineered bacteria (Fig. S3b). The S-ACP activity in the control group containing the empty vector of *Bacillus subtilis* was a mere 52 nmol/d/g, while the S-ACP activity of the engineered bacteria expressing these four genes reached as high as 6028 nmol/d/g, 7811 nmol/d/g, and 11,267 nmol/d/g, respectively. Compared to the control group inoculated with *Bacillus subtilis* containing an empty vector, the available phosphorus content in the soil treated with these four engineered strains was significantly increased, with the available phosphorus content increasing to 3.74 mg/kg, 2.78 mg/kg, 2.83 mg/kg, and 2.82 mg/kg, respectively. These outcomes robustly confirm the positive influence of the engineered bacteria on elevating the soil’s bioavailable phosphorus content.

Subsequently, these genes were transferred into *Bacillus subtilis* for further validation, and it was found that the S-ACP activity of these four recombinant strains also increased (Fig. 3b), leading to a significant enhancement in the content of available phosphorus in the soil (Fig. 3c). This discovery prompted us to further screen for four phosphatase genes within PSM16 that are implicated in the dephosphorylation function. We constructed a phylogenetic tree (Fig. 3d) incorporating four identified phosphatase proteins—834, 917, 925, and 974—alongside ten previously reported phosphatase proteins to elucidate their evolutionary relationships. The results revealed that protein 834 exhibits a close phylogenetic relationship with the phosphatase protein sequence NP_001340555.1 from soybean (*Glycine max*), supported by a bootstrap value of 61%. Proteins 917 and 925 also clustered with protein 834, indicating their close evolutionary affinity with the soybean phosphatase, with bootstrap values of 47% and 60%, respectively. In contrast, protein 974 showed a closer relationship with the phosphatase protein sequence NP_009651.3 from *Saccharomyces cerevisiae*, supported by a bootstrap value of 38%. Furthermore, these proteins displayed more distant phylogenetic relationships with phosphatase proteins from other sources, such as A*rabidopsis thaliana, Penicillium chrysogenum, Methylocella silvestris, Serratia* sp*., Pseudomonas aeruginosa,* and *Aspergillus melleus*. This finding suggests that although these proteins may share a common ancestral origin, they have undergone significant divergence during evolution, potentially leading to unique functional or structural characteristics.

Furthermore, we aim to conduct an in-depth investigation into the mechanisms of these genes and to explore their practical application potential in agricultural production. Our objective is to contribute significantly to the enhancement of crop yields and the amelioration of the soil environment, thereby fostering sustainable agricultural practices.

### Effect of different phosphatases on the germination rate of *Arabidopsis thaliana* seeds

**Fig 4:**
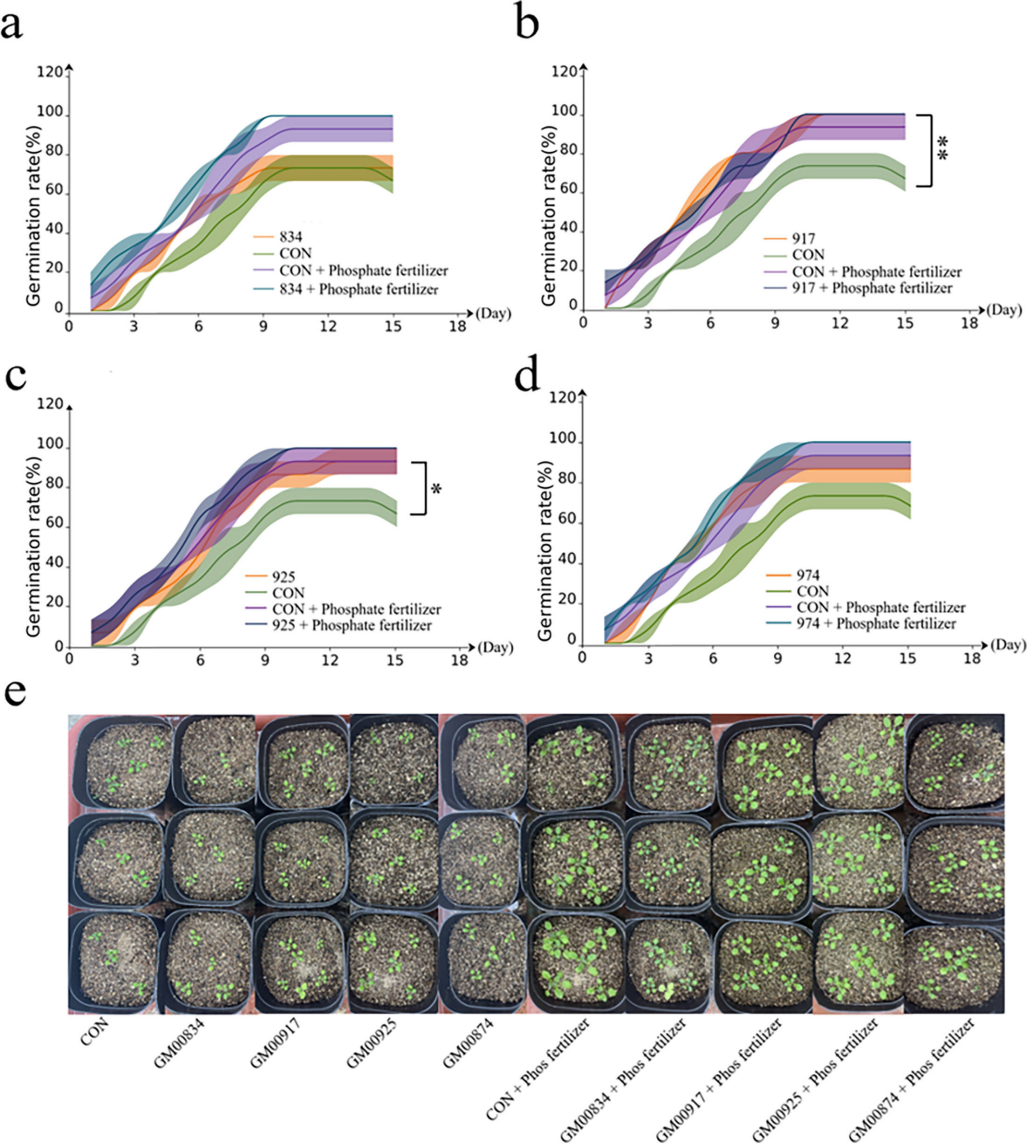
Effect of different phosphatases on the germination rate of *Arabidopsis thaliana* seeds. The germination rate of *Arabidopsis thaliana* after adding the recombinant bacteria including the expression plasmids of GM00834 (a), GM00917 (b), GM00925 (c), and GM00974 (d). (e) The control image in Fig. 4e is the same as the control image in Fig. 2b as they are derived from the same experimental batch. This repetition reflects the consistency of the experimental conditions and is not an error. Germinated *Arabidopsis thaliana* after adding recombinant bacteria on day 15. Results are presented as mean values ± SD. *N* = 3. Statistical significance determined by *t*-test. *P < 0.05, **P < 0.01, and ***P < 0.001.

In subsequent experiments, *Bacillus subtilis* transformed with genes of GM000834, GM000917, GM000925, and GM000974 were introduced into the soil for *Arabidopsis thaliana* cultivation. The result demonstrated a pronounced increase in the germination rate of the treated *Arabidopsis thaliana*, with the rate stabilizing around the tenth day of the experiment. The control group (CON) exhibited a germination rate of 66.67%, whereas the groups treated with the engineered bacteria, corresponding to the GM000834, GM000917, GM000925, and GM000974 genes, showed accelerated germination, culminating in final germination rates of 73.3%, 100%, 93.3%, and 86.7%, respectively (Fig. 4a through d). Remarkably, upon the application of phosphorus fertilizer, the germination rates in all treatment groups with the engineered bacteria increased to 100% (Fig. 4e). This underscores the engineered bacteria’s capacity to enhance seed germination and growth; the phosphate-solubilizing function of PSM16 can promote seed germination.

These findings offer a profound insight into the specific impacts of the heterologous expression of engineered bacteria on *Arabidopsis* cultivation. The results are instrumental in advancing agricultural generation technology and hold significant promise for boosting crop yields.

### Dephosphorylated phosphatase in pig manure and biogas residue composts

**Fig 5:**
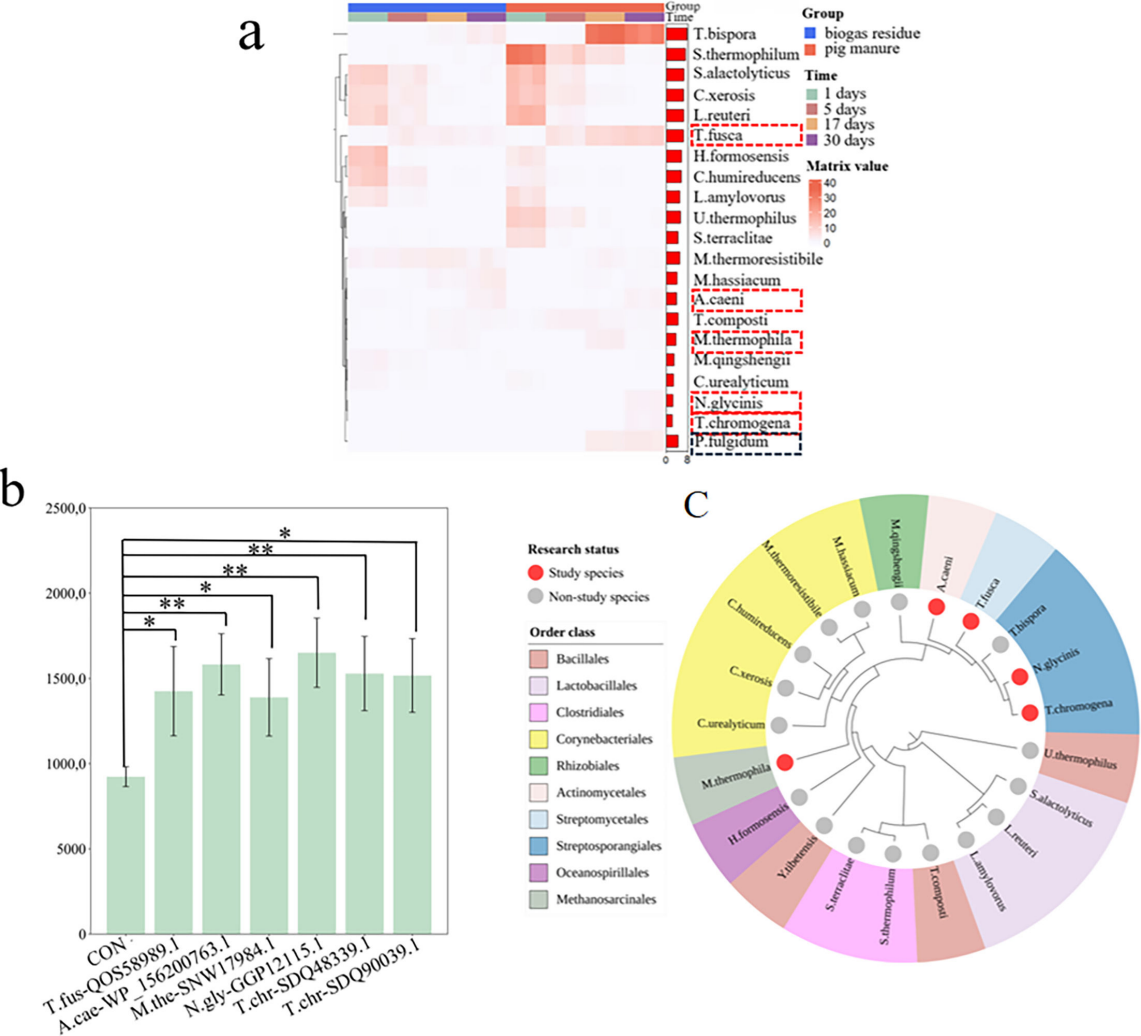
Identification and analysis of phosphatase-producing bacterial strains in compost samples. (**a**) Abundance of strains at various time intervals in the compost, with red markers indicating the abundance of homologous phosphatase strains identified through screening and black markers indicating that the acid phosphatase activity is negative. (**b**) Phosphatase activity in *Bacillus subtilis* strains heterologously expressing the homologous phosphatase enzyme. (**c**) A phylogenetic tree representing 21 strains found in the compost, with red markers highlighting the strains examined in this study.

Compost is recognized for its ability to enhance soil fertility. However, in our analysis of the metagenomic samples from the SRP328020 data set, which encompasses pig manure, the presence of the PSM16 strain was not detected. Despite this, we identified 41 species among the 885 detected species within the data set that possessed 94 homologous genes similar to the dephosphorylation gene of PSM16. These genes were classified into four orthogroups. In this study, we examined 21 bacterial strains selected for their significant abundance in the SRP328020 data set. The heatmaps (Fig. 5a) illustrate the distribution of these strains based on data obtained from SRP328020 at various time intervals. Typically, as compost matures, its temperature increases, potentially exceeding 60°C. Consequently, in this study, we selected six thermostable strains for assessment of dephosphorylated phosphatase activity (Table S3). Five of these strains, carrying six genes from two orthogroups, demonstrated dephosphorylated phosphatase activity (Fig. 5b), including *Thermobifida fusca* (T.fus-QOS58989.1), *Actinotalea ceani* (A.cae-WP_156200763.1), *Mycolicibacyerium thermoresistibile* (M.the-SNW17984.1), *Nonomuraea glycinis* (N.gly-GGP12115.1), and *Thermostaphylospra chromogena* (T.chr-SDQ48339.1, T.chr- SDQ90039.1).

We constructed a phylogenetic tree based on the homologous genes identified in the 21 bacterial strains, with the strains under investigation distinctly marked in red (Fig. 5c). Our analysis revealed that bacteria capable of secreting phosphatase enzymes are widespread across various bacterial orders. Furthermore, we identified a significant correlation between the level of phosphatase activity and the phylogenetic relatedness of the strains. This correlation suggests that strains exhibiting greater evolutionary divergence (i.e., a wider phylogenetic distance) tend to display lower phosphatase activity.

## DISCUSSION

The study has further corroborated the capacity of PSM16 to solubilize phosphorus. The findings indicate that PSM16 can markedly improve the efficiency of phosphorus utilization within the environment and significantly elevate the levels of bioavailable phosphorus in the soil, which in turn stimulates seed germination. The principal mechanism by which PSM16 achieves phosphorus solubilization is through the secretion of phosphatase enzymes. The utilization of PSM16 as a biofertilizer has the potential to notably decrease the reliance on conventional phosphorus fertilizers, thereby reducing input costs and the environmental footprint. Additionally, it enhances soil biological activity and crop yields, contributing to relieving ecological and environmental concerns.

Other research (2, 7) often emphasizes an equally significant mechanism of phosphorus solubilization by PSB: the secretion of organic acids like gluconic acid, lactic acid, citric acid, and oxalic acid. This process typically leads to a reduction in the pH of the fermentation medium. This study further revealed that PSM16 generates a diverse range of organic acids that effectively reduce soil pH. This reduction not only optimizes the soil pH for enhanced plant growth but also facilitates the chelation of various metal cations. As a result, the soil’s electrical conductivity is modified, leading to a multifaceted enhancement in soil fertility.

Moreover, additional research has demonstrated that PSB can enhance seed germination and exhibit other plant growth-promoting rhizobacteria (PGPR) effects. These effects include the secretion of plant growth-promoting hormones (PGPHs) (24) such as indole-3-acetic acid (IAA) and gibberellins (GA). To investigate whether PSM16 could stimulate plant growth through PGPR effects beyond its phosphate-solubilizing capabilities, we conducted a comprehensive analysis of its entire genome. Our analysis did not reveal genes associated with the production of PGPHs like IAA, nor did we identify genes involved in cytokinin synthesis. However, we did discover genes responsible for the synthesis of siderophores, which are known to promote plant growth, as well as genes encoding nitrogenase. Additionally, we found genes that contribute to plant stress resistance, including those for antioxidant enzymes and genes involved in the synthesis of antibiotics. These latter genes may protect plants by combating pathogens. This study was primarily concerned with the phosphate-solubilizing functions of PSM16 and did not experimentally explore or confirm the roles of other PGPR-related genes.

PSB holds great promise in agricultural applications owing to their unique prophylactic functions (25, 26). Currently, no investigation on the dephosphorylating function of *Pediococcus pentosaceus* has been conducted. PSM16 (19) is a *Pediococcus pentosaceus* strain isolated from sow milk, and *Pediococcus pentosaceus* is one of the commonly used fermentation agents in the fermentation industry, especially in fermented dairy products. *Lactobacillus strains* are commonly employed as expression host bacteria, and food-grade gene expression systems have been successfully integrated into certain strains such as *Lactobacillus casei* (27), *Lactobacillus swissii* (28), and *Pediococcus pentosaceus* (29). Among these, *Pediococcus pentosaceus* stands out as one of the most promising strains due to its representativeness (30), extensive research value, and depth of study. Transforming it into a bacteriophage offers the advantage of safety and harmlessness.

Currently, known PSB strains are predominantly categorized within three phyla: *Thick-walled Bacteria, Actinobacteria*, and *Ascomycetes* (31, 32). At the genus level, strains belonging to the genera *Pseudomonas, Bacillus,* and *Rhizobium* are generally considered to be the most viable P-enriching solvents (3). In contrast to these, our study has identified PSM16, a strain of *Pediococcus pentosaceus* not naturally occurring, which exhibits the potential to serve as an agricultural phosphate-solubilizing agent. The deployment of PSM16 as a biofertilizer in agriculture necessitates an examination of its interactions with indigenous soil microorganisms and its impact on the rhizosphere microbiota. Consequently, future research should concentrate on assessing the practical feasibility of PSM16 as an agricultural application.

In this study, we conducted a comprehensive whole-genome sequencing analysis to identify phosphatase-related genes within PSM16. These genes were then heterologously expressed in *Bacillus subtilis* to evaluate the strain’s capacity to produce phosphatase. Our efforts culminated in the successful identification of key phosphatase-related genes: GM000834, GM000917, GM000925, and GM000974 in PSM16. These discoveries significantly bolster the investigation into the microbial mechanisms underlying phosphorus solubilization for agricultural use.

Moreover, based on the analysis of bioinformatics, we identified the homologs of PSM16 phosphatase in compost and confirmed their phosphatase activity post-heterologous expression. Understanding the phosphatase activity of different strains and their phylogenetic relationships can help us select microbial strains with efficient phosphorus-solubilizing capabilities for use in agriculture and environmental remediation. This not only validated our bioinformatics approach but also offered a pragmatic strategy for screening microorganisms capable of phosphorus solubilization. These insights enrich our comprehension of microbial communities and their metabolic roles within compost and introduce novel candidate strains and proteins for the innovation of biofertilizers aimed at enhancing soil phosphorus utilization.

### Conclusions

In this study, we delved into the intrinsic mechanisms of phosphorus solubilization by PSM16 and explored its role in enhancing soil fertility and seed germination, thereby uncovering the substantial potential of PSM16 in increasing phosphorus bioavailability. Our findings indicate that PSM16 is endowed with phosphorus-solubilizing capabilities that can enrich soil quality, notably elevate the levels of bioavailable phosphorus in the soil, enhance phosphorus utilization, and stimulate both plant germination and growth. Its application in conjunction with phosphorus fertilizers can increase the efficiency of fertilizer use, decrease chemical fertilizer consumption, and foster the sustainable advancement of agriculture—a strategy that embodies high efficiency, eco-friendliness, and economic viability.

Through whole-genome sequencing, we identified 13 phosphatase-related genes within PSM16. These genes were heterologously expressed in *Bacillus subtilis* to ascertain the acid phosphatase activity of the engineered strains. The impact on soil’s bioavailable phosphorus content was evaluated by treating soil with these strains. Ultimately, we successfully identified four genes that exhibited phosphatase activity: the genes of GM1000834, GM1000917, GM1000925, and GM1000974. Additionally, combined with bioinformatics, we detected homologous phosphatases present in compost.

Thus, the outcomes of this research can provide promising candidate strains for the development of efficient bio-organic fertilizers, enriching the repertoire of tools for sustainable agricultural practices.

## MATERIALS AND METHODS

### Strains, plasmids, and reagents

The strains and plasmids used are listed in Table S4. PSM16 was a strain of *Pediococcus pentosaceus* isolated from porcine milk in our previous study (19). The Whole Genome Shotgun project of PSM16 has been deposited at GenBank under the accession number JBIWYI000000000.1. Primers were synthesized by Sangon Biotech Co., Ltd. (Shanghai, China) (Table S5).

Phosphorus-free nutrient solution: 0.85 g/L ammonium nitrate, 0.95 g/L potassium nitrate, 0.14701 g/L calcium chloride dihydrate, 0.185 g/L magnesium sulfate heptahydrate, 0.415 mg/L potassium iodide, 3.1 mg/L borate, 11.15 mg/L molybdenum sulfate tetrahydrate, 4.3 mg/L zinc sulfate heptahydrate, and 0.125 mg/L sodium permanganate dihydrate, 0.0125 mg/L copper sulfate pentahydrate, 0.125 mg/L cobalt chloride hexahydrate, 18.65 mg/L disodium iminodiacetic acid, 1.39 mg/L ferrous sulfate heptahydrate, 970 mL of mono-distilled water, with the pH adjusted to 5.8, and 10 g sucrose.

Nutrient solution with added phosphorus: 0.34 g/L potassium dihydrogen phosphate was added to the phosphate-free nutrient solution.

Vermiculite: A mineral that contains a trace amount of the inorganic phosphorus compound P_2_O_5._ Its chemical composition is represented by the formula (Mg,Fe)_3_(Al,Si)_4_O_10_(OH)_2_·4H_2_O.

Phosphate fertilizer: Calcium superphosphate is used, which contains 12% P_2_O_5_, P1 denotes the application of a low phosphate fertilizer content at 75 mg/kg, while P2 signifies the use of a high phosphate fertilizer content at 300 mg/kg.

### Determination of effective phosphorus

Two milliliters of PSM16 was added to 15 g of vermiculite, followed by the addition of 20 mL of mono-distilled water and mixed well, no PSM16 as a control group, and three parallel groups. The mixtures were placed in a constant temperature incubator at 37°C for 24 hours. The samples were evenly placed on the air-drying disk to form a thin layer of 2–3 cm, and timely crushing and turning operations were performed to remove debris. The samples were crushed, thoroughly mixed, sieved through a 1 mm soil sieve, mixed again, and then placed in milled bottles. The effective phosphorus content of the specimens was determined using the sodium hydrogen carbonate solution-Mo-Sb anti-spectrophotometric method (33).

### Determination of total phosphorus

PSM16 bacterial solution (1.9 mL) was added to 15 g of vermiculite and mixed with 20 mL of mono-distilled water, set up without bacterial solution as a control group, and set up three parallel groups. The total phosphorus content of the samples was determined by the alkali fusion-molybdenum antimony anti-spectrophotometric method (34).

### Determination of acid phosphatase activity

The strains were cultured in an MRS liquid medium for 12 hours at 37°C, 900 rpm. Three groups were set up in parallel, and samples from the culture were taken to determine the acid phosphatase-producing activity of the strains by colorimetric assay using the Soil Acid Phosphatase (S-ACP) Activity Kit obtained from Sangon Biotech Co., Ltd.

### Strain SCFA determination

Individual clones were incubated at 37°C on an orbital shaker set to 900 rpm for durations of 0 h, 24 h, 48 h, and 72 h. Subsequently, the cultures were centrifuged at 8,000 rpm for 5 minutes to pellet the cells. The resulting supernatant was then treated with 25% metaphosphoric acid at 4°C for 3 hours, using a supernatant-to-metaphosphoric acid ratio of 9:1. Following this, the mixture was centrifuged again at 15,000 rpm for 10 minutes, and the supernatant was passed through a 0.45 µm filter membrane. Each treatment was replicated three times independently. Short-chain fatty acids (SCFAs) were analyzed using a high-performance gas chromatograph fitted with a DB-FFAP column (30 m × 250 μm × 0.25 µm), with nitrogen as the carrier gas at a flow rate of 0.8 mL/min, a split ratio of 50:1, and an injection volume of 1 µL. The temperatures for the oven, detector, and injector were set to 220°C, 280°C, and 250°C, respectively. The concentration of SCFAs was determined using the external standard curve method, employing standard solutions of acetate, propionate, butyrate, isobutyrate, isovalerate, and valerate for calibration.

### *Arabidopsis* planting

Seeds were poured into EP tubes filled with sterile water and placed into a refrigerator at 4°C for 3 days for vernalization. Before planting *Arabidopsis thaliana*, the vermiculite was sterilized, mixed with bacterial culture, set up a control group without bacteria, and it was placed in an incubator at 37°C for 24 h. After cooling, the vermiculite was packed into square boxes and placed in red trays. Lay the seeds flat on the weighing paper, and point the seeds with the gun tip with vermiculite. We conducted our experiments with a consistent set of controls. The control images used in Fig. 2b and 4e are identical, reflecting the same experimental batch used for both sets of experiments.

### Construction of engineering bacteria

Competent cells of *Bacillus subtilis* 168 (35) were prepared using the direct induction transformation method (36). Specifically, a single colony was picked from *Bacillus subtilis 168*, inoculated into an appropriate amount of LB liquid medium, and cultured aerobically overnight at 37°C and 900 rpm/min. On the following day, 50 µL of overnight bacterial culture was transferred to 1.5 mL of LB medium, and incubation was continued at 37°C until the OD_600_ value was about 1.5, which took about 3.5–4 h. Eighty microliters of 20% xylose solution was added to make a final concentration of 1%, and the temperature was adjusted to 30°C to continue the incubation for 2 h. Bacteria were collected by centrifugation, the supernatant was removed, 200 µL of LB non-antibiotic liquid medium was added to suspend the bacteria, and 10–20 µL of plasmid was added. The mixture was incubated at 30°C for 1 h, and 100 µL was spread on plates containing 10 µg/mL kanamycin and incubated overnight in a 37°C incubator.

### Phylogenetic tree construction

A set of ten previously characterized phosphatase proteins were obtained from GenBank, with accession numbers as follows: NP_009651.3 (37), KAK1140980.1 (38), NP_001340555.1 (39), AAA33693.1 (40), ACK51488.1 (41), OHQ66101.1 (42), AEG28968.1 (43), AAO24567.1 (44), AWL09029.1 (45), and AED93912.1 (46), respectively, from *Saccharomyces cerevisiae* S288C, *Aspergillus melleus*, *Glycine max, Penicillium chrysogenum*, *Methylocella silvestris* BL2, *Pseudomonas aeruginosa*, *Serratia* sp. AS13, *Arabidopsis thaliana*, *Aquirufa nivalisilvae*, and *Arabidopsis thaliana*. We constructed a phylogenetic tree including the phosphatase proteins 834, 917, 925, and 974 along with them by using the Maximum Parsimony (MP) model of software MEGA-X.

### Bioinformatics analysis

To investigate the phosphate-solubilizing bacteria in soil, data with the accession number SRP328020 (47) were collected, encompassing metagenomic sequencing data from pig manure and biogas residue. Subsequently, MetaPhlAn 4 was utilized for bacterial species detection (48), which identified 885 species within the SRP328020 data set (47). The genomes, coding DNA sequences (CDS), and proteins of these detected species were retrieved from the NCBI. Furthermore, blastp (49) was employed to detect homologous sequences among the phosphatase-related proteins from *Pediococcus pentosaceus* PSM16, with the reference genome GCF_000014505.1 (50).

Estimation of species abundances was conducted through a multi-step process: i) Reads were mapped to the detected species using BWA (Burrows-Wheeler Aligner) (51). ii) The Transcripts Per Million (TPM) for phosphatase-related genes were calculated based on the count of mapped paired reads for these target genes. iii) The TPM values were normalized relative to the total number of reads mapped to the detected species in each sample. iv) Heatmaps illustrating the abundance data were generated utilizing the ComplexHeatmap package (52).

The construction of a phylogenetic tree for species containing phosphatase-related genes was a multi-step process: i) Initially, Orthofinder was utilized to identify orthogroups among the protein sequences of all species involved (53). ii) Subsequently, each orthogroup was aligned using MAFFT, applying a maximum of 1,000 iterations for accurate alignment (54). iii) Following alignment, IQ-TREE was employed to construct the phylogenetic tree, also with up to 1,000 iterations to ensure robust phylogenetic inference (55). iv) The constructed tree was then visualized and further enhanced using the Interactive Tree Of Life (iTOL) tool (56).

For the localization of scaffolds related to the PSM16 phosphatase: i) BEDTOOLS was first used to extract and convert the coding DNA sequence (CDS) data of PSM16 and *Pediococcus pentosaceus* (PPEN) into BED format files (57). ii) Subsequently, the synteny analysis was performed using JCVI, with a conservation score (cscore) threshold set at 0.99 to ensure high similarity, which facilitated the precise determination of scaffold localization for the PSM16 phosphatase (58).

## SUPPLEMENTAL MATERIAL

Supplemental figures spectrum.00491-25-s0001.pdf Figures S1 to S3.

Supplemental tables spectrum.00491-25-s0002.docx Tables S1 to S5.

ASM does not own the copyrights to Supplemental Material that may be linked to, or accessed through, an article. The authors have granted ASM a non-exclusive, world-wide license to publish the Supplemental Material files. Please contact the corresponding author directly for reuse.
