# Supplementary material for: Phosphate-solubilizing function of Pediococcus pentosaceus PSM16 and its underlying mechanism
Source: Microbiol Spectr. 2025 Jun 10;13(7):e00491-25. doi: 10.1128/spectrum.00491-25 (PMC12252388; doi:10.1128/spectrum.00491-25)
Supplement: Supplemental figures — Figures S1 to S3. [file spectrum.00491-25-s0001.pdf]

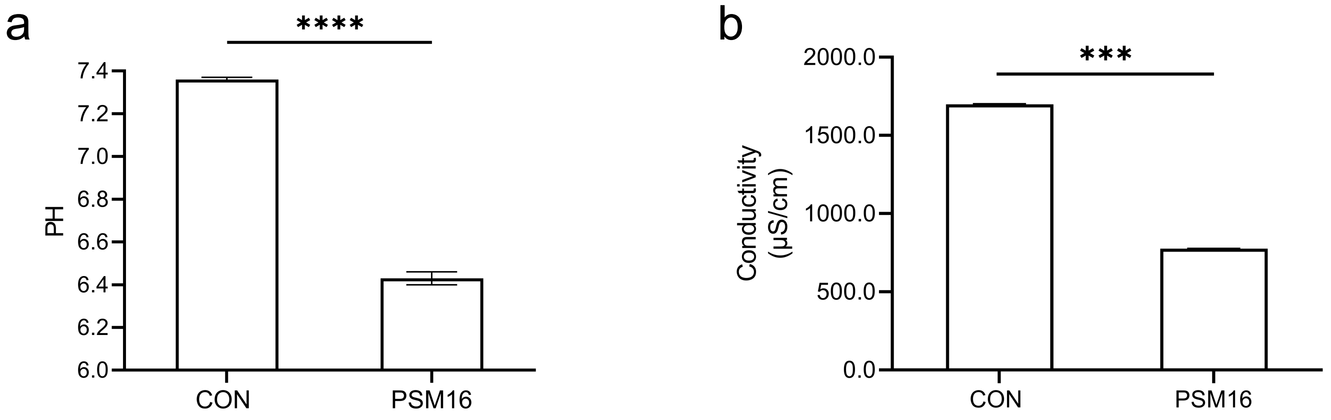

Supplementary Figure 1. Impact of PSM16 on Soil Conductivity and pH.
(a) Influence of PSM16 on soil electrical conductivity. (b) Impact of PSM16 on soil pH levels. Data are presented as mean $\pm$ standard deviation (SD). *$P < 0.05$, **$P < 0.01$, ***$P < 0.001$, determined by t-test.

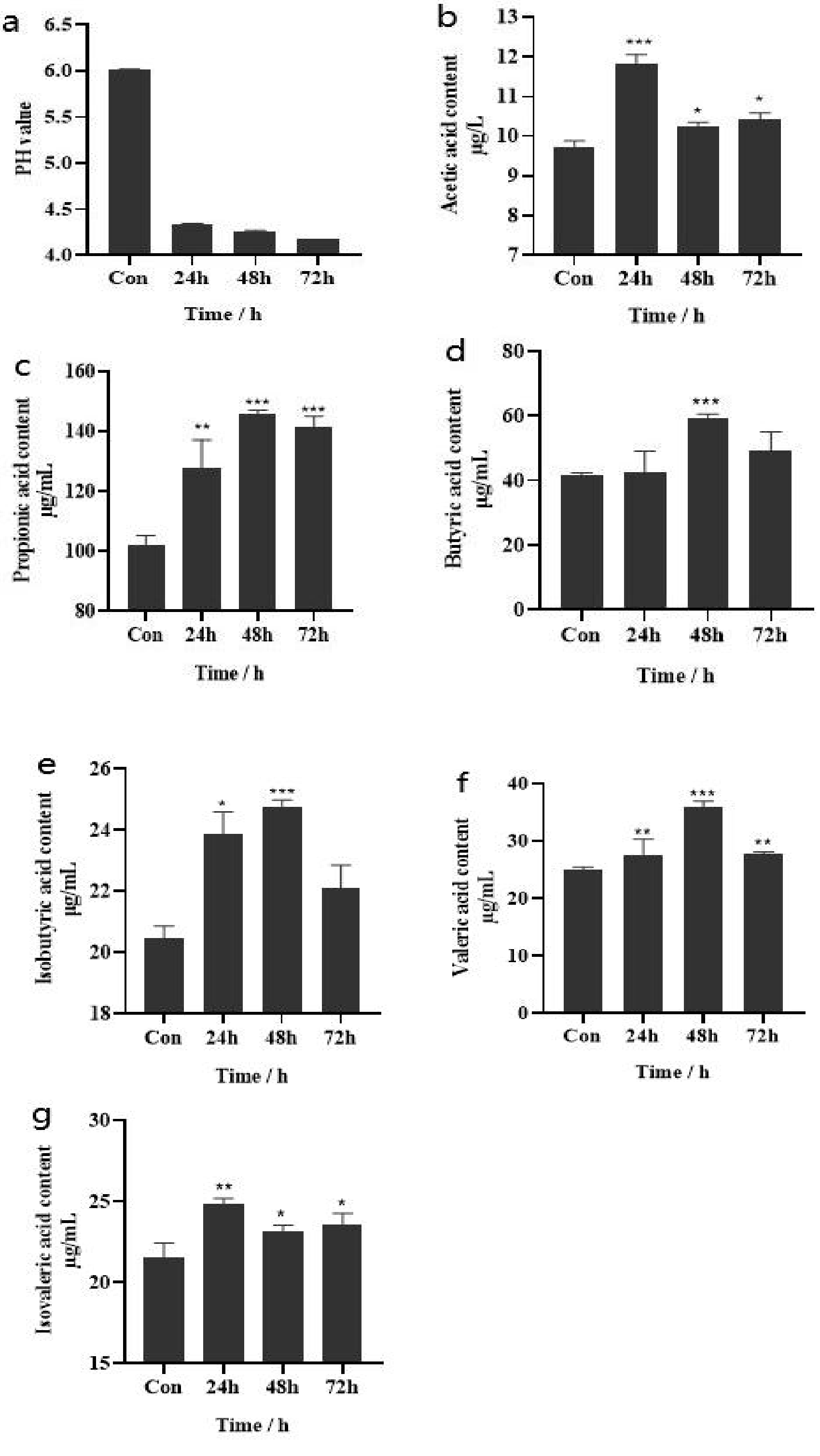

Supplementary Fig 2. SCFAs of *P. pentosaceus* PSM16.
(a) Acid production curve of PSM16 with incubation time, Acetic acid production curve of PSM16 with time. (b) Propionic acid production curve. (c) Butyric acid production curve (d) Isobutyric acid production curve (e) Valeric acid production curve (f) and isovaleric acid production curve (g) Data are expressed as mean ± standard deviation (SD). *P < 0.05, **P < 0.01, ***P < 0.001, as determined by t-tests.

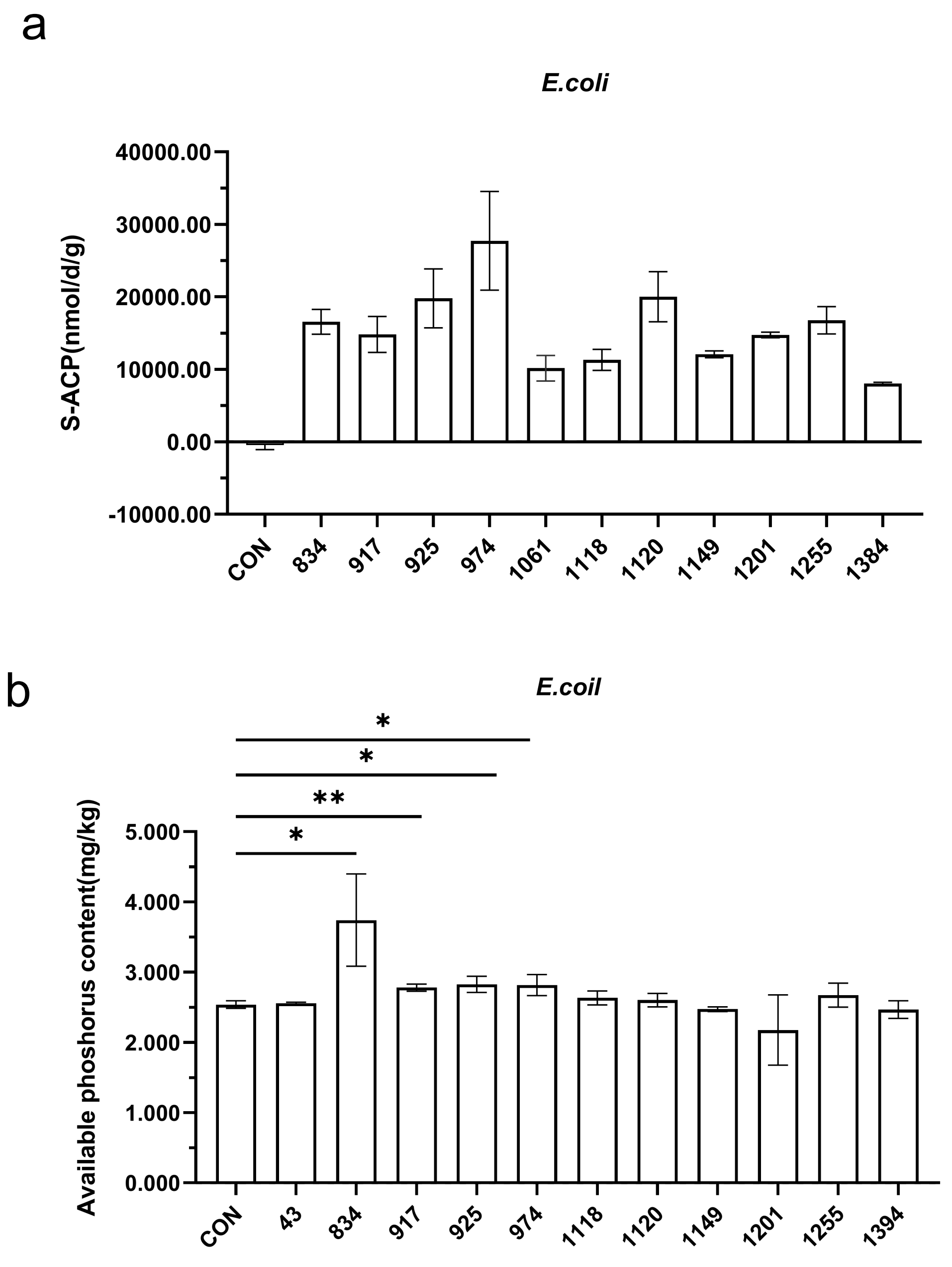

Supplementary Fig 3. Impact of Recombinant Bacteria on Soil Available Phosphorus Content.

(a) S-ACP activity levels in individual recombinant *E. coli* strains. (b) Changes in soil available phosphorus content following the introduction of the recombinant *E. coli* strains. Data are expressed as mean ± standard deviation (SD). *P < 0.05, **P < 0.01, ***P < 0.001, as determined by t-tests.
