# Supplementary material for: Phosphate-solubilizing function of Pediococcus pentosaceus PSM16 and its underlying mechanism
Source: Microbiol Spectr. 2025 Jun 10;13(7):e00491-25. doi: 10.1128/spectrum.00491-25 (PMC12252388; doi:10.1128/spectrum.00491-25)
Supplement: Supplemental tables — Tables S1 to S5. [file spectrum.00491-25-s0002.docx]

Table S1. Enzymatic Activities of *P. pentosaceus* PSM16

| Numble | Ennzyme | Result | Score |
| --- | --- | --- | --- |
| 1 | Control | - | - |
| 2 | Alkaline | - | - |
| 3 | Esterase(C4) | + | 1 |
| 4 | Lipoesterase(C8) | + | 1 |
| 5 | Lipoidase(C14) | + | 2 |
| 6 | Leucine arylaminase | + | 5 |
| 7 | Vaine arylaminase | + | 4 |
| 8 | Cystine arylaminase | + | 2 |
| 9 | Trypsin | + | 1 |
| 10 | Chymotrypsin | - | - |
| 11 | Acid phosphate | + | 4 |
| 12 | Naphthol-AS-BI-phosphohydrola | + | 5 |
| 13 | α-galactosidase | + | 1 |
| 14 | β- galactosidase | - | - |
| 15 | β-glucuronidase | - | - |
| 16 | α-glucosidase | + | 1 |
| 17 | β- glucosidase | + | 1 |
| 18 | N-Acetylglucosaminase | + | 0.5 |
| 19 | α-mannosidase | - | - |
| 20 | β-fucosodase | - | - |

Note: The scoring system, ranging from 0 to 5, corresponds to the intensity of color depth, with 0 indicating no depth, 5 signifying the most profound depth, and scores of 2 to 4 reflecting moderate intensities. The color depths can be translated into approximate milligram molecule concentrations (nM) as follows: a score of 1 equates to a release of 5 nM, a score of 2 to 10 nM, a score of 3 to 20 nM, a score of 4 to 30 nM, and a score of 5 to 40 nM or greater.

Table S2. Phosphatase Genes Investigated in This Study

| Name | Description | Accession | Query Cover | Identities |
| --- | --- | --- | --- | --- |
| GM000043 | histidine phosphatase family protein | WP_011673535.1 | 99% | 100.00% |
| GM000551 | Putative phosphatase | ARW20081.1 | 99% | 99.63% |
| GM000834 | Asp23/Gls24 family envelope stress response protein | WP_002832905.1 | 91% | 100.00% |
| GM000917 | phosphoglycolate phosphatase | AZP91607.1 | 98% | 73.43% |
| GM000925 | sugar-phosphatase | WP_201244996.1 | 99% | 100.00% |
| GM000974 | exopolyphosphatase | KRN47720.1 | 99% | 100.00% |
| GM0001061 | Putative phosphatase YcsE | ARW20381.1 | 99% | 100.00% |
| GM0001118 | histidine phosphatase family protein | WP_201245242.1 | 99% | 100.00% |
| GM0001120 | histidine phosphatase family protein | WP_023440024.1 | 99% | 100.00% |
| GM0001149 | response regulator transcription factor | WP_002834460.1 | 99% | 100.00% |
| GM0001201 | hypothetical protein | WP_286121246.1 | 98% | 100.00% |
| GM0001255 | CpsB/CapC family capsule biosynthesis tyrosine phosphatase | WP_094104871.1 | 99% | 100.00% |
| GM0001384 | phosphatase PAP2 family protein | WP_167399724.1 | 99% | 99.56% |

Table S3. Protein Sequences of Identified Homologous Phosphatases

| Strains | Protein Description | Protein Sequence ID |
| --- | --- | --- |
| *Planigilum fulgidum* | pyrophosphatase PpaX | SFG37440.1 |
| *Thermobifida fusca* | HAD family phosphatase | QOS58989.1 |
|  | Ppx/GppA phosphatase family protein | QOS60012.1 |
| *Actinotalea ceani* | Ppx/GppA phosphatase family protein | WP_156200763.1 |
| *Mycolicibacyerium thermoresistibile* | HAD family hydrolase | SNW17984.1 |
| *Nonomuraea glycinis* | Ppx/GppA phosphatase family protein | GGP12115.1 |
| *Thermostaphylospra chromogena* | Ppx/GppA phosphatase family protein | SDQ48339.1 |
|  | HAD family phosphatase | SDQ90039.1 |

Table S4. List of Plasmids and Strains Employed in This Study

| Plasmid and strain | Description | References or sources |
| --- | --- | --- |
| PSM16 | 1. *pentosaceus* | (Zhou et al., 2021) |
| KT2440 | *Pseudomonas putida* | (An & Moe, 2016) |
| *Bacillus subtilis* 168 | Expression vector | (Fu et al., 2010) |
| pP43NMK-P43-043-km | histidine phosphatase family protein | This study |
| pP43NMK-P43-551-km | Putative phosphatase | This study |
| pP43NMK-P43-834-km | Asp23/Gls24 family envelope stress response protein | This study |
| pP43NMK-P43-917-km | phosphoglycolate phosphatase | This study |
| pP43NMK-P43-925-km | sugar-phosphatase | This study |
| pP43NMK-P43-974-km | exopolyphosphatase | This study |
| pP43NMK-P43-1061-km | Putative phosphatase YcsE | This study |
| pP43NMK-P43-1118-km | histidine phosphatase family protein | This study |
| pP43NMK-P43-1120-km | histidine phosphatase family protein | This study |
| pP43NMK-P43-1149-km | response regulator transcription factor | This study |
| pP43NMK-P43-1201-km | hypothetical protein | This study |
| pP43NMK-P43-1255-km | CpsB/CapC family capsule biosynthesis tyrosine phosphatase | This study |
| pP43NMK-P43-1384-km | phosphatase PAP2 family protein | This study |

Table S5. Primers Utilized for Amplification in This Study

| Primer | Primer sequence (5′-3′) | Application |
| --- | --- | --- |
| GM000043 | ACGAAATTATATTTTATTAG | histidine phosphatase family protein |
| GM000551 | ACTCCCAAATTAATTGCTAT | Putative phosphatase |
| GM000834 | GATAAAGTAGCACAACCAGA | Asp23/Gls24 family envelope stress response protein |
| GM000917 | ACTAAGTATAAAGTTGCTTT | phosphoglycolate phosphatase |
| GM000925 | AGTATAAAATTAATCGCCAT | sugar-phosphatase |
| GM000974 | AAAAATTTGGTAATAGTAGA | exopolyphosphatase |
| GM0001061 | GATATTGATGGAACCTTAAT | Putative phosphatase YcsE |
| GM0001118 | AAAACCGTCAATTATTATTT | histidine phosphatase family protein |
| GM0001120 | AAAAAATTAAAACTATATTT | histidine phosphatase family protein |
| GM0001149 | TCAAAGACGATATTAGTTGT | response regulator transcription factor |
| GM0001201 | ATGATTAAATATGCAATTTT | hypothetical protein |
| GM0001255 | ATGAACTTAATTGATTTACA | CpsB/CapC family capsule biosynthesis tyrosine phosphatase |
| GM0001384 | TTAAAAGATACTGATAATAT | phosphatase PAP2 family protein |
